# Surgical resection of an isolated superficial temporal artery aneurysm

**DOI:** 10.1016/j.jvscit.2023.101361

**Published:** 2023-10-30

**Authors:** Jake L. Rosen, Archana Babu, Mohammed Irfan Ali, Michael J. Nooromid

**Affiliations:** a Sidney Kimmel Medical College, Thomas Jefferson University, Philadelphia, PA; b Department of Surgery, Thomas Jefferson University, Philadelphia, PA; c Division of Vascular and Endovascular Surgery, Department of Surgery, Thomas Jefferson University, Philadelphia, PA

**Keywords:** Aneurysmal pathology, Temporal artery aneurysms

## Abstract

In the present report, we describes a case of surgical resection of an isolated superficial temporal artery aneurysm without underlying systemic pathology. Although aneurysms of this sort most commonly occur in the setting of recent trauma, this case demonstrates an uncommon presentation. We hope to further contribute to the literature regarding this condition.

Superficial temporal artery aneurysms (STAAs) are uncommon presentations typically developing after trauma or surgery and revealed as pseudoaneurysms.^1^^,^^2^ Previous STAAs in the absence of trauma have rarely been described in the literature, with some noted coincidentally with intracranial aneurysms.^3^ Isolated STAAs without comorbid conditions appear to be exceedingly rare. We report the case of a 73-year-old female patient presenting with this pathology. The patient provided written informed consent for the report of her case details and imaging details.

## Case report

**Fig 1:**
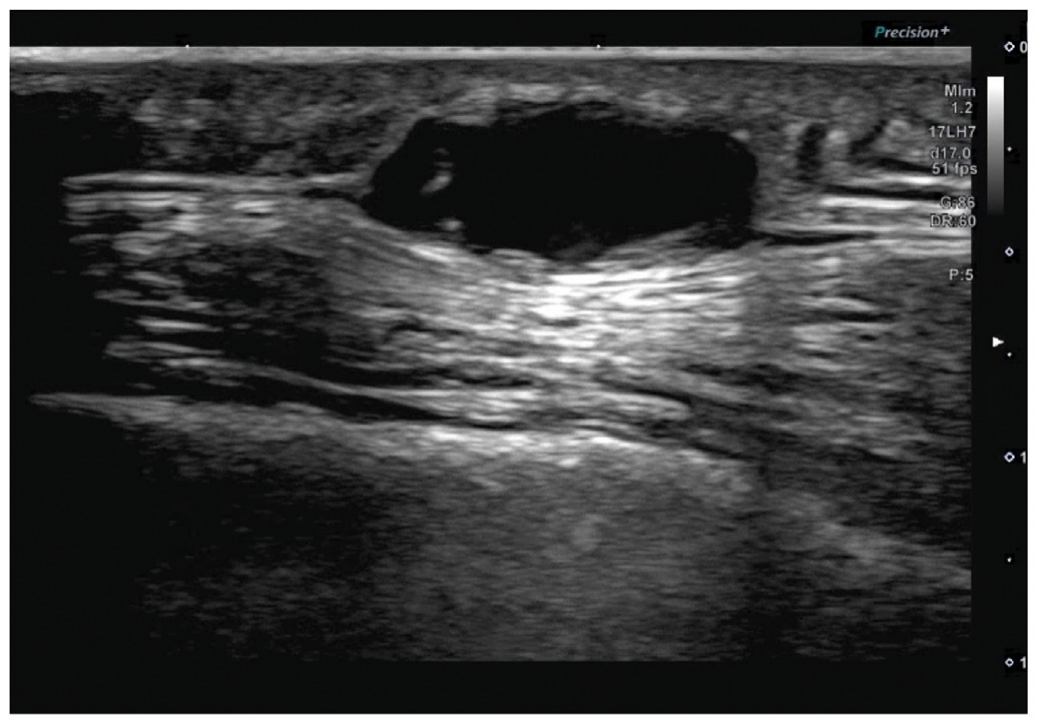
Aneurysm visualized on ultrasound.

A 73-year-old woman with a medical history of essential hypertension, stage 3A chronic kidney disease, and pre-diabetes mellitus presented to vascular surgery via referral from her primary care physician regarding an asymptomatic right-sided pulsatile mass located on her scalp. Ultrasound performed before the vascular surgery referral revealed a right STAA located within the frontal branch and measuring 0.80 by 0.50 cm (Fig 1). In-office vascular physical examination revealed no other appreciable aneurysms in the extremities or the abdomen. The decision was made to surgically resect the aneurysm.

**Fig 2:**
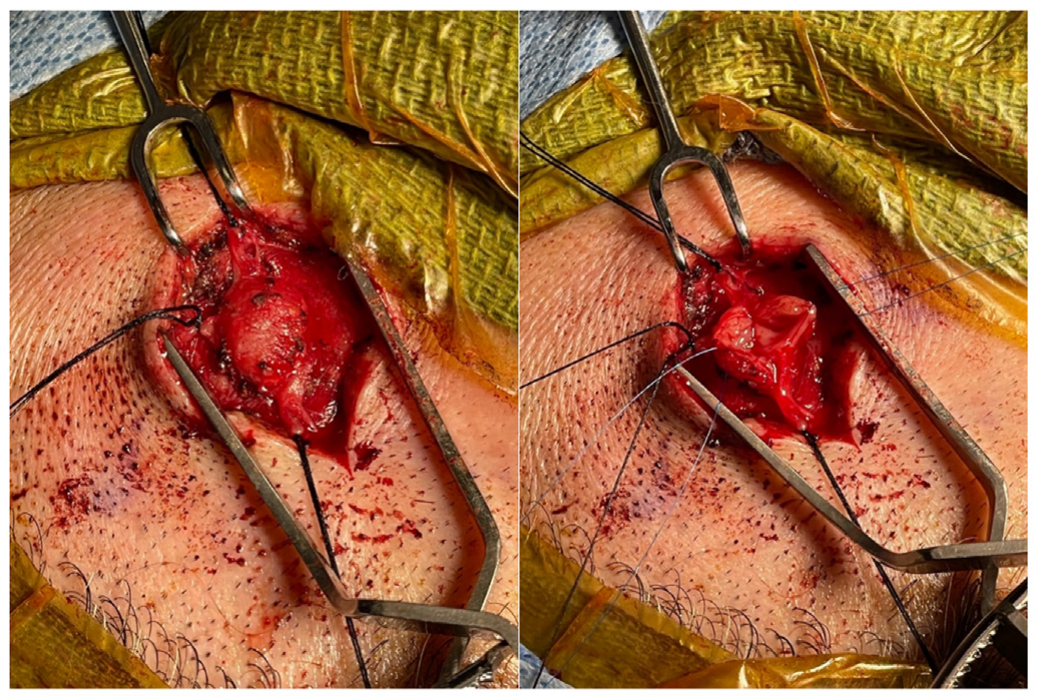
Aneurysm identification and excision.

Intraoperative ultrasound revealed similar aneurysmal characteristics. An oblique incision was made over the STAA. The incision was placed in the line of the superficial temporal artery, with dissection using a scalpel and electrocautery. Once the aneurysm was circumferentially located and isolated, the cephalad, caudad, and lateral feeding vessels were also isolated and tied off using 4-0 silk suture (Fig 2). After confirming the loss of pulsatility within the aneurysmal area, we resected the specimen and sent it for further examination. With hemostasis confirmed, we closed the incision with 3-0 Vicryl and 4-0 Monocryl suture (Ethicon). The patient was discharged the same day.

Further workup of the case included pathologic examination and imaging studies. Pathologic examination revealed chronic medial fibrosis and inflammation with intimal hyperplasia, consistent with aneurysmal dilation. Computed tomography angiography showed no other significant stenosis in any of the aortic arch or cerebral vessels. No other comorbid aneurysm was present in the aortoiliac distribution, and the abdominal ultrasound findings were negative for pathology. The erythrocyte sedimentation rate was mildly elevated at 66 mm/h, and the C-reactive protein level was within normal limits. All other laboratory test results revealed no abnormalities.

## Discussion

This case represents a rare pathology that is not extensively reported in the literature. Much of the literature surrounding STAAs is in the setting of recent trauma. However, these aneurysms are typically pseudoaneurysms. Pseudoaneurysms, or false aneurysms, are very different from true aneurysms. The pathophysiology of a pseudoaneurysm means that there is no layer of the arterial wall formed in the bulge. Rather, the collective outpouching is a result of the clotting cascade initiated.^4^ Much of the literature surrounding STAAs and pseudoaneurysms discusses the pathology resulting from trauma, citing the cause in ≤95% of cases.^5^ It appears that STA pseudoaneurysms are quite rare, accounting for ∼400 to 500 cases in the literature and representing 1%of all aneurysms.^6^

However, isolated STAAs are much rarer. A detailed case series of six patients reported by Pipinos et al^7^ revealed that only one of their operations was in the absence of trauma. Kim et al^8^ studied 12 patients with STAAs presenting with a pulsating mass and no neurologic deficits. Although the differential diagnosis for this condition includes degenerative, congenital, traumatic (and iatrogenic) etiologies and concomitant connective tissue disease or vasculitis, truly isolated cases appear to be sparse in the literature.^7^ In a review spanning the literature for the previous 150 years, van Uden et al^9^ noted only 20 of the 160 STAA cases were true aneurysms.

Given the rare nature of true STAAS, the pathophysiology has yet to be fully understood. Much like other aneurysms present throughout the body, atherosclerosis, aging, wall stiffening, and underlying congenital abnormalities are all possible etiologies and have been proposed by others.^10^ In our case, we confirmed aneurysmal wall degeneration through postoperative histologic imaging. Furthermore, we performed follow-up imaging using computed tomography and laboratory studies to assess the possibility for the aneurysm to be secondary to another medical condition.

The procedure itself is quite quick, simple, and without much technical difficulty. Technical details have been described in previous reports of pseudoaneurysm repair; the same procedural techniques apply for true aneurysm repair.^6^ Although rare, complications of ligation of the superficial temporal artery include infection, facial nerve injury, skin necrosis, and secondary hemorrhage. An important aspect of the operation is locating the aneurysm and excising its margins. Intraoperative ultrasound is helpful in discerning the precise location of the aneurysm. Without active pulsation, gross inspection could be inadequate in assessing aneurysm’s location.

## Conclusions

Surgical resection for an isolated STAA is an exceedingly rare procedure of an already uncommon location for vascular pathology. We hope this case adds to the small body of reported work on this medical condition.

## Disclosures

None.
